# Supplementary material for: Unlocking Phytate with Phytase: A Meta-Analytic View of Meat-Type Chicken Muscle Growth and Bone Mineralization Potential
Source: Animals (Basel). 2024 Jul 17;14(14):2090. doi: 10.3390/ani14142090 (PMC11274156; doi:10.3390/ani14142090)
Supplement: Supplementary file 1 [file animals-14-02090-s001.zip › animals-3071972-supplementary.pdf]

**Supplementary Material**

Unlocking Phytate with Phytase: A Meta-Analytic View of Meat-type Chicken Muscle Growth and Bone Mineralization Potential.

Emmanuel Nuamah [1,*], Utibe Mfon Okon [2], Eungyeong Jeong [1,†], Yejin Mun [1,†], Inhyeok Cheon [1,†], Byungho Chae [1,†], Fred-erick Nii Ako Odoi [3], Dong-wook Kim [4], Nag-Jin Choi [1,*]

Contents:

Supplementary Figures S1-S4 (*Starter Growth Performance*)

Supplementary Figures S5-S8 (*Grower-Finisher Growth Performance*)

Supplementary Figures S9-S12 (*Starter Bone Strength and Mineralization*)

Supplementary Figures S13-S16 (*Grower-Finisher Bone Strength and Mineralization*)

Supplementary Figure S17. Trim and Fill funnel plot assessment for testing and adjusting publication bias on starter broilers' growth outcomes

Supplementary Figure S18. Trim and Fill funnel plot assessment for testing and adjusting publication bias on grower-finisher broilers' growth outcomes

Supplementary Figure S19. Trim and Fill funnel plot assessment for testing and adjusting publication bias on starter broilers' bone strength and mineralization outcomes

Supplementary Figure S20. Trim and Fill funnel plot assessment for testing and adjusting publication bias on grower-finisher broilers' bone strength and mineralization outcomes

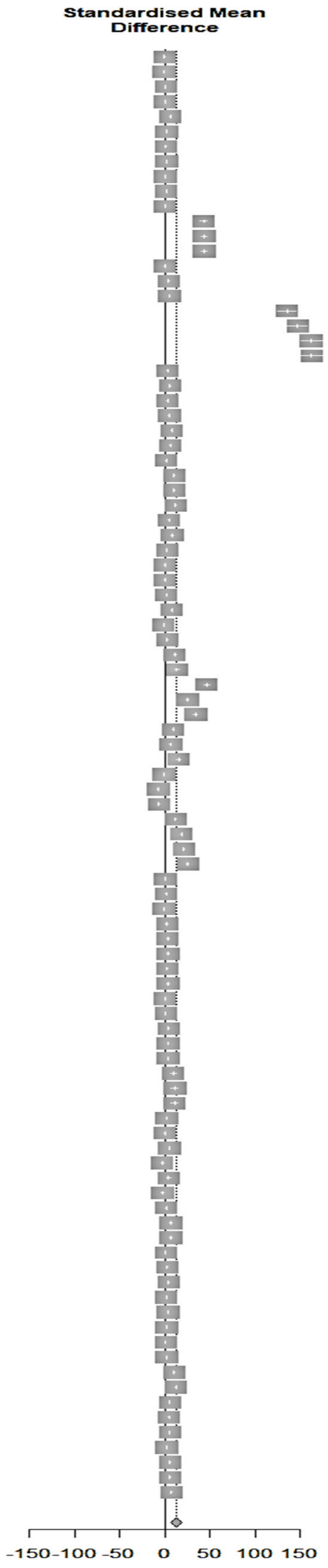


| Study | SMD | 95%-CI |
|---|---|---|
| Ajuwon et al. 2018 | -0.27 | [ -0.63; 0.09] |
| Ajuwon et al. 2018.1 | -1.19 | [ -1.57; -0.80] |
| Beeson et al. 2017 | 0.97 | [ 0.61; 1.34] |
| Beeson et al. 2017.1 | 0.25 | [ -0.10; 0.60] |
| Borda-Molina et al. 2016 | 6.09 | [ 5.48; 6.69] |
| Bowen et al. 2022 | 2.30 | [ 2.07; 2.53] |
| Bowen et al. 2022.1 | 1.20 | [ 1.00; 1.39] |
| Bowen et al. 2022.2 | 2.40 | [ 2.16; 2.63] |
| Bowen et al. 2022.3 | 0.50 | [ 0.32; 0.68] |
| Bowen et al. 2022.4 | 1.60 | [ 1.39; 1.80] |
| Bowen et al. 2022.5 | 0.10 | [ -0.08; 0.28] |
| Broch et al. 2020 | 43.05 | [ 39.84; 46.26] |
| Broch et al. 2020.1 | 43.52 | [ 40.28; 46.77] |
| Broch et al. 2020.2 | 43.52 | [ 40.28; 46.77] |
| Broch et al. 2018 | -0.07 | [ -0.27; 0.14] |
| Broch et al. 2018.1 | 4.10 | [ 3.74; 4.46] |
| Broch et al. 2018.2 | 5.39 | [ 4.95; 5.84] |
| Campasino et al. 2014 | 135.57 | [125.94; 145.21] |
| Campasino et al. 2014.1 | 147.20 | [136.74; 157.67] |
| Campasino et al. 2014.2 | 162.36 | [150.82; 173.91] |
| Campasino et al. 2014.3 | 162.95 | [151.37; 174.54] |
| Cowieson et al. 2006 | 2.93 | [ 2.35; 3.51] |
| Cowieson et al. 2006.1 | 5.84 | [ 4.90; 6.77] |
| Cowieson et al. 2006.2 | 3.31 | [ 2.69; 3.94] |
| Cowieson et al. 2006.3 | 4.98 | [ 4.16; 5.80] |
| Cowieson et al. 2006.4 | 7.57 | [ 6.41; 8.73] |
| Cowieson et al. 2006.5 | 6.01 | [ 5.05; 6.96] |
| Cowieson et al. 2013 | 1.49 | [ 1.18; 1.81] |
| Dersjant-Li et al. 2020 | 10.19 | [ 9.46; 10.92] |
| Dersjant-Li et al. 2020.1 | 10.45 | [ 9.71; 11.20] |
| Dersjant-Li et al. 2020.2 | 11.89 | [ 11.05; 12.73] |
| Dersjant-Li et al. 2022 | 4.91 | [ 4.21; 5.61] |
| Dersjant-Li et al. 2022.1 | 8.27 | [ 7.19; 9.36] |
| Dessimoni et al. 2019 | 2.59 | [ 2.34; 2.85] |
| Ennis et al. 2020 | 0.00 | [ -0.25; 0.25] |
| Ennis et al. 2020.1 | 0.00 | [ -0.25; 0.25] |
| Ennis et al. 2020.2 | 1.81 | [ 1.51; 2.12] |
| Gehring et al. 2014 | 8.00 | [ 7.42; 8.58] |
| Gehring et al. 2014.1 | -1.26 | [ -1.47; -1.05] |
| Ghahri et al. 2012 | 2.71 | [ 2.15; 3.27] |
| Ghahri et al. 2012.1 | 10.79 | [ 9.18; 12.40] |
| Ghahri et al. 2012.2 | 13.66 | [ 11.64; 15.67] |
| Gulizia et al. 2022 | 46.40 | [ 43.45; 49.36] |
| Gulizia et al. 2022.1 | 25.00 | [ 23.40; 26.60] |
| Gulizia et al. 2022.2 | 34.28 | [ 32.09; 36.46] |
| Hernandez et al. 2022 | 9.26 | [ 8.02; 10.51] |
| Houshyar et al. 2023 | 6.70 | [ 5.89; 7.50] |
| Houshyar et al. 2023.1 | 15.26 | [ 13.54; 16.98] |
| Javadi et al. 2022 | -1.00 | [ -1.30; -0.71] |
| Javadi et al. 2022.1 | -7.28 | [ -8.06; -6.50] |
| Javadi et al. 2022.2 | -6.40 | [ -7.10; -5.70] |
| Jlali et al. 2024 | 11.83 | [ 11.14; 12.52] |
| Jlali et al. 2024.1 | 18.49 | [ 17.43; 19.56] |
| Jlali et al. 2024.2 | 21.23 | [ 20.02; 22.45] |
| Jlali et al. 2024.3 | 25.77 | [ 24.29; 27.24] |
| Jozefiak et al. 2010 | 0.82 | [ 0.60; 1.04] |
| Karami et al. 2020 | 1.66 | [ 1.30; 2.02] |
| Karami et al. 2020.1 | -1.11 | [ -1.44; -0.77] |
| Kiarie et al. 2015 | 2.42 | [ 1.21; 3.63] |
| Kiarie et al. 2015.1 | 3.18 | [ 1.78; 4.58] |
| Kiarie et al. 2015.2 | 3.86 | [ 2.27; 5.45] |
| Kiarie et al. 2015.3 | 2.88 | [ 1.56; 4.21] |
| Kiarie et al. 2015.4 | 3.60 | [ 2.09; 5.12] |
| Kriseldi et al. 2021 | 0.00 | [ -0.18; 0.18] |
| Kriseldi et al. 2021.1 | 1.09 | [ 0.89; 1.28] |
| Kriseldi et al. 2021.2 | 4.27 | [ 3.94; 4.59] |
| Kriseldi et al. 2021.3 | 3.74 | [ 3.45; 4.04] |
| Kriseldi et al. 2021.4 | 3.74 | [ 3.45; 4.04] |
| Kwon et al. 2022 | 9.51 | [ 6.12; 12.89] |
| Kwon et al. 2022.1 | 11.15 | [ 7.22; 15.08] |
| Kwon et al. 2022.2 | 11.01 | [ 7.13; 14.90] |
| Liu et al. 2016 | 2.26 | [ 1.99; 2.52] |
| Powell et al. 2008 | 0.00 | [ -0.23; 0.23] |
| Ptak et al. 2015 | 5.04 | [ 4.47; 5.61] |
| Ptak et al. 2013 | -2.88 | [ -4.20; -1.56] |
| Ptak et al. 2013.1 | 4.39 | [ 2.65; 6.14] |
| Ptak et al. 2013.2 | -2.37 | [ -3.57; -1.18] |
| Shang et al. 2015 | 1.43 | [ 0.86; 2.00] |
| Shi et al. 2023 | 6.84 | [ 5.89; 7.79] |
| Shi et al. 2023.1 | 6.61 | [ 5.68; 7.53] |
| Walk et al. 2018 | 0.94 | [ 0.56; 1.32] |
| Walk et al. 2018.1 | 2.63 | [ 2.13; 3.12] |
| Walk et al. 2018.2 | 4.11 | [ 3.47; 4.75] |
| Walk et al. 2013 | 1.69 | [ 1.50; 1.88] |
| Walk et al. 2013.1 | 3.68 | [ 3.41; 3.95] |
| Walk et al. 2013.2 | 2.19 | [ 1.98; 2.39] |
| Walk and Olukosi 2019 | 0.90 | [ 0.66; 1.13] |
| Walk and Olukosi 2019.1 | 2.29 | [ 2.00; 2.58] |
| Walk and Poernama 2019 | 10.49 | [ 9.86; 11.11] |
| Walk and Poernama 2019.1 | 12.59 | [ 12.07; 13.11] |
| Walk et al. 2014 | 5.49 | [ 5.10; 5.88] |
| Walk et al. 2014.1 | 4.66 | [ 4.31; 5.01] |
| Walk et al. 2014.2 | 5.49 | [ 5.10; 5.88] |
| Woyengo et al. 2010 | 2.39 | [ 1.92; 2.86] |
| Zhang et al. 2022b | 5.64 | [ 5.13; 6.16] |
| Zhang et al. 2022b.1 | 5.72 | [ 5.20; 6.24] |
| Zhang et al. 2022b.2 | 7.38 | [ 6.73; 8.03] |
| **Random effects model** | **12.57** | **[ 6.61; 18.54]** |

Heterogeneity: $I^2 = 100\%$, $\tau^2 = 895.4718$
Test for overall effect: $p < 0.01$

Figure S1. Forest plot of the effect size or standardized mean difference and 95% confidence interval of phytase on starter broilers' average daily feed intake (ADFI). The solid vertical black line represents the mean difference between zero and no effect. Points to the left of the solid vertical black line represent a reduction in ADFI, while points to the right indicate an increase in ADFI.

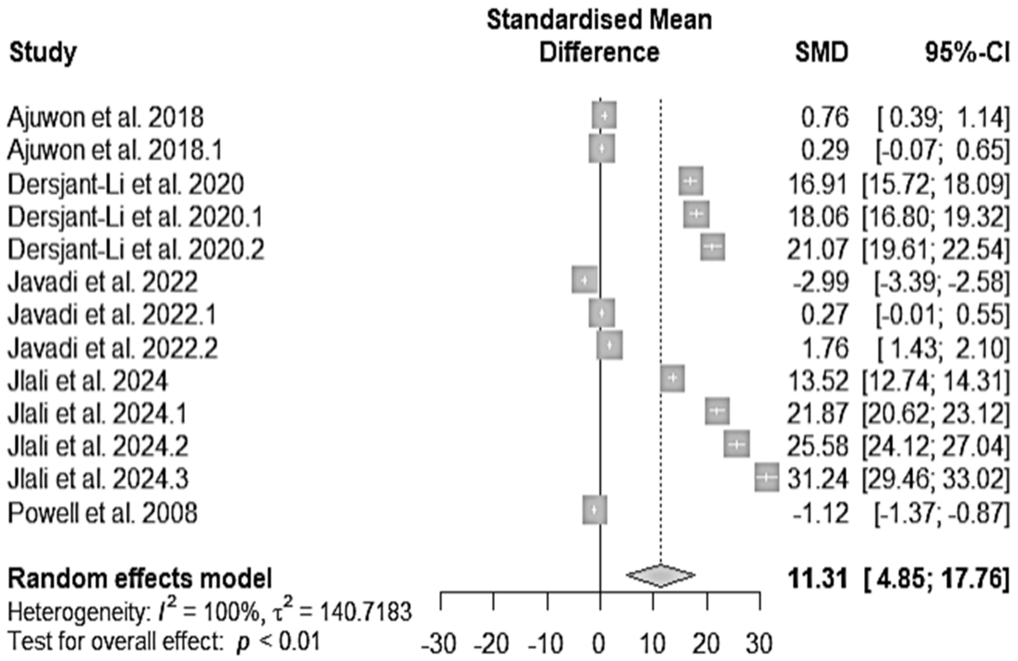


Figure S2. Forest plot of the effect size or standardized mean difference and 95% confidence interval of phytase on starter broilers' average daily gain (ADG). The solid vertical black line represents the mean difference between zero and no effect. Points to the left of the solid vertical black line represent a reduction in ADG, while points to the right indicate an increase in ADG.

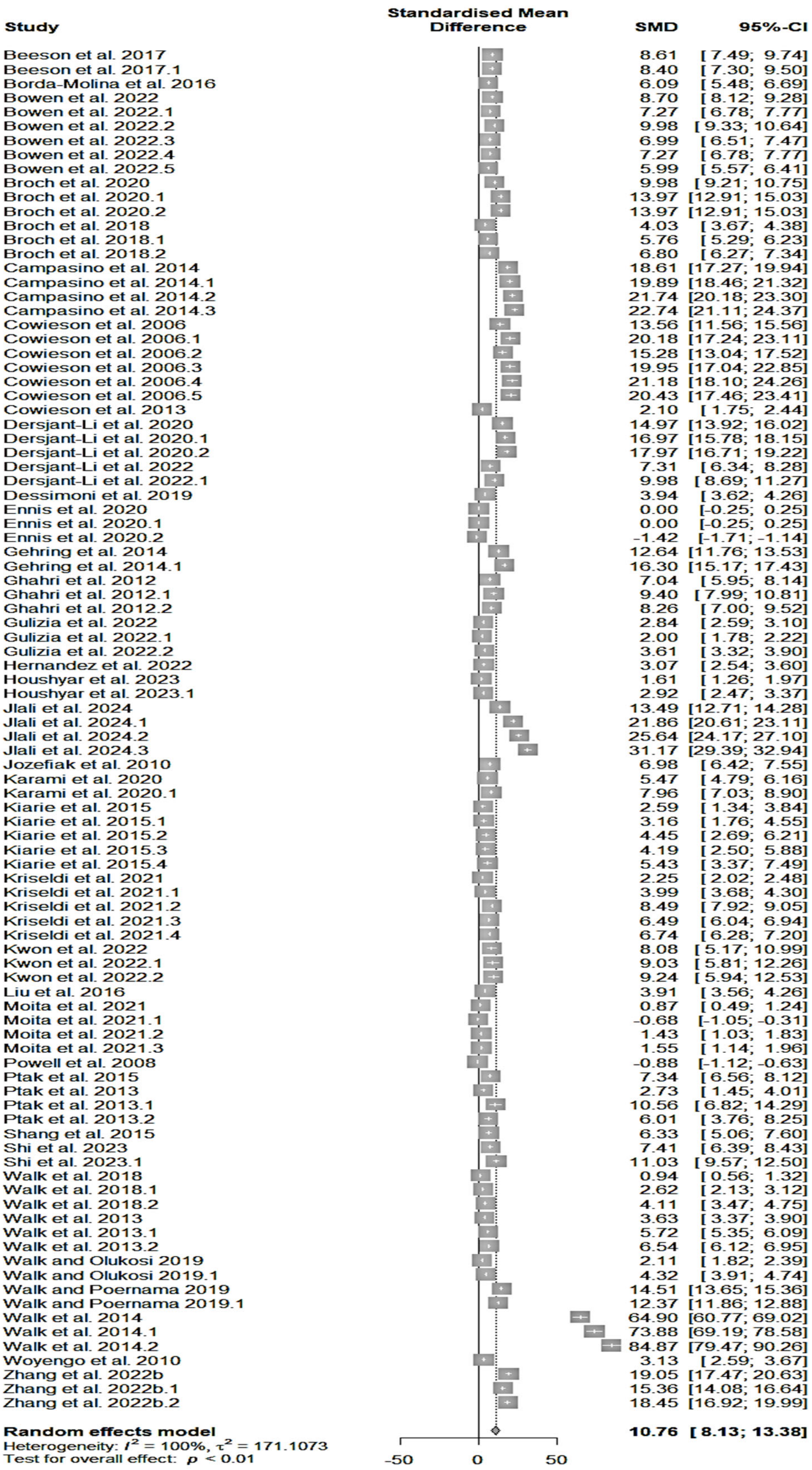


Figure S3. Forest plot of the effect size or standardized mean difference and 95% confidence interval of phytase on starter broilers' body weight gain (BWG). The solid vertical black line represents the mean difference between zero and no effect. Points to the left of the solid vertical black line represent a reduction in BWG, while points to the right indicate an increase in BWG.

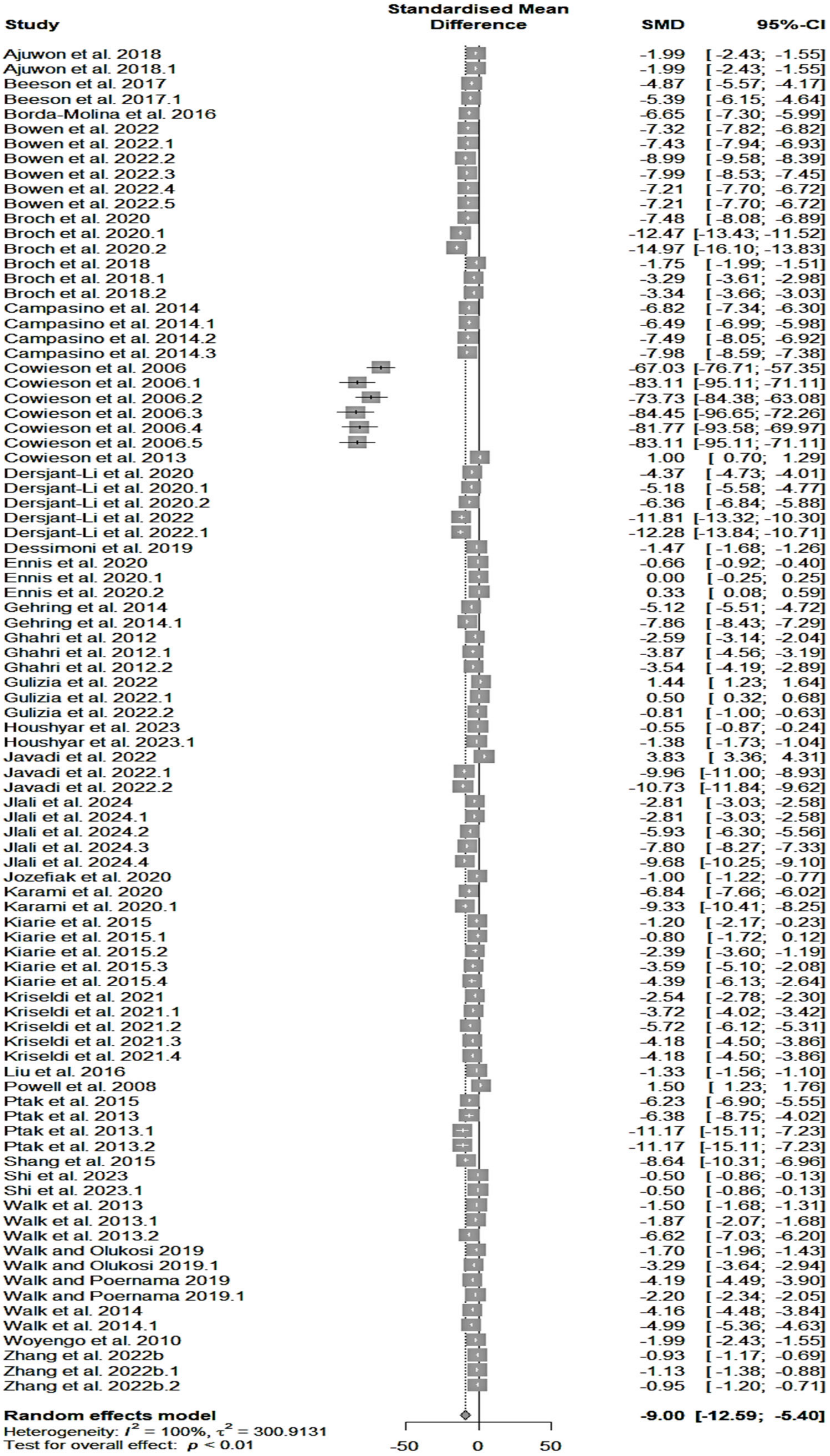


Figure S4. Forest plot of the effect size or standardized mean difference and 95% confidence interval of phytase on starter broilers' feed conversion ratio (FCR). The solid vertical black line represents the mean difference between zero and no effect. Points to the left of the solid vertical black line represent a reduction in FCR, while points to the right indicate an increase in FCR.

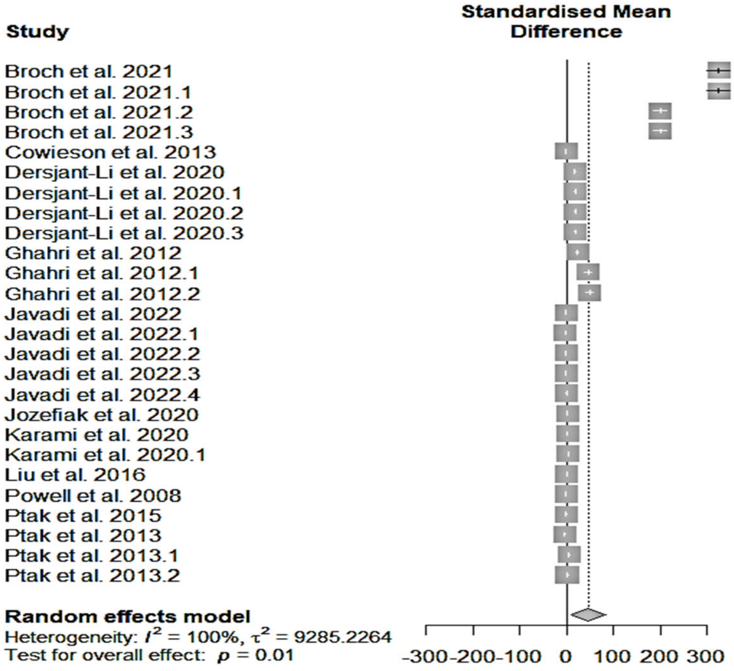


Figure S5. Forest plot of the effect size or standardized mean difference and 95% confidence interval of phytase on grower-finisher broilers' average daily feed intake (ADFI). The solid vertical black line represents the mean difference between zero and no effect. Points to the left of the solid vertical black line represent a reduction in ADFI, while points to the right indicate an increase in ADFI.

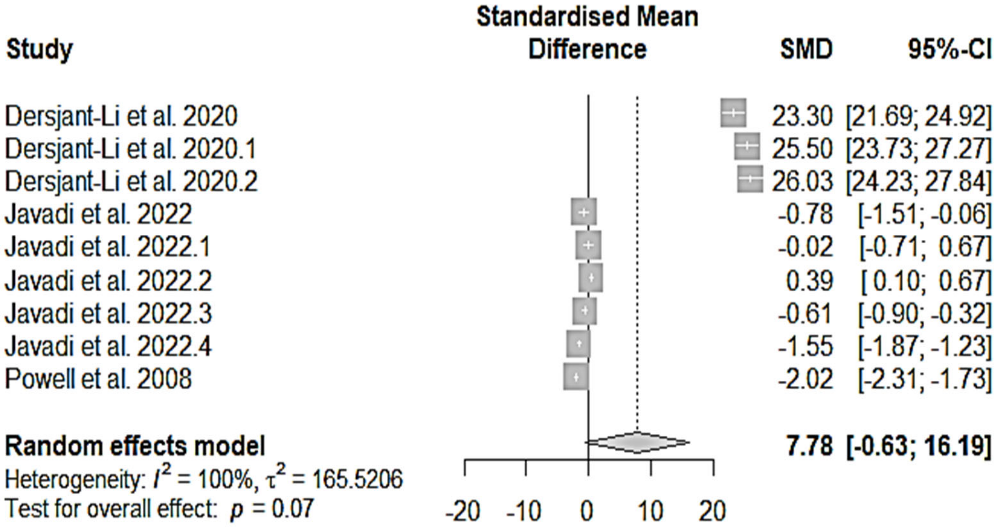


Figure S6. Forest plot of the effect size or standardized mean difference and 95% confidence interval of phytase on grower-finisher broilers' average daily gain (ADG). The solid vertical black line represents the mean difference between zero and no effect. Points to the left of the solid vertical black line represent a reduction in ADG, while points to the right indicate an increase in ADG.

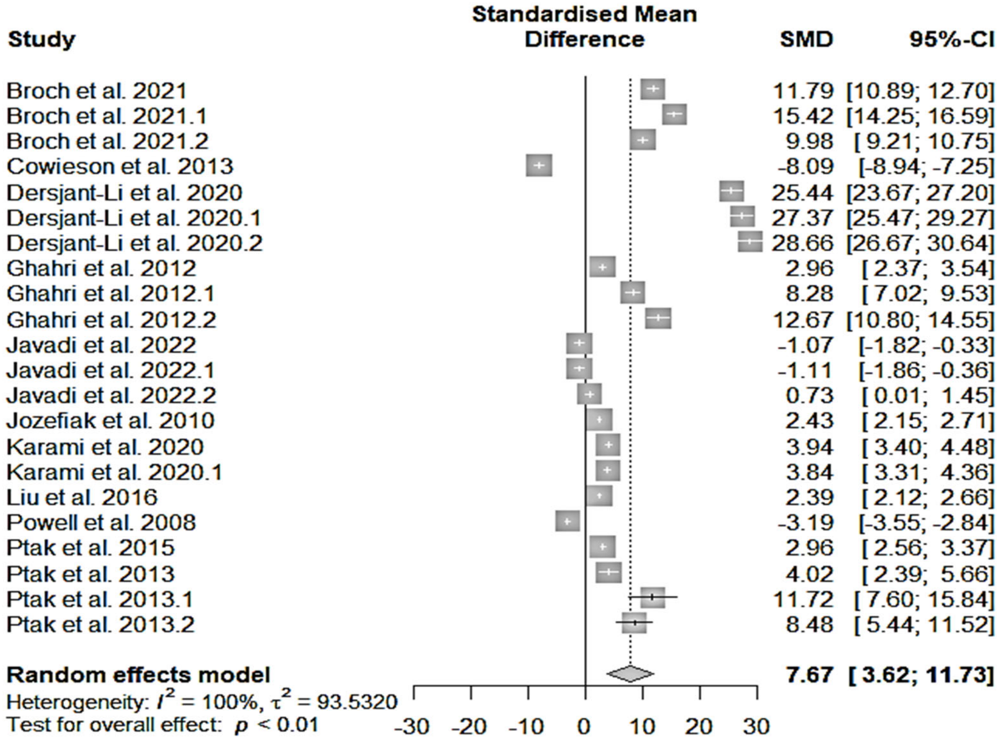


Figure S7. Forest plot of the effect size or standardized mean difference and 95% confidence interval of phytase on grower-finisher broilers' body weight gain (BWG). The solid vertical black line represents the mean difference between zero and no effect. Points to the left of the solid vertical black line represent a reduction in BWG, while points to the right indicate an increase in BWG.

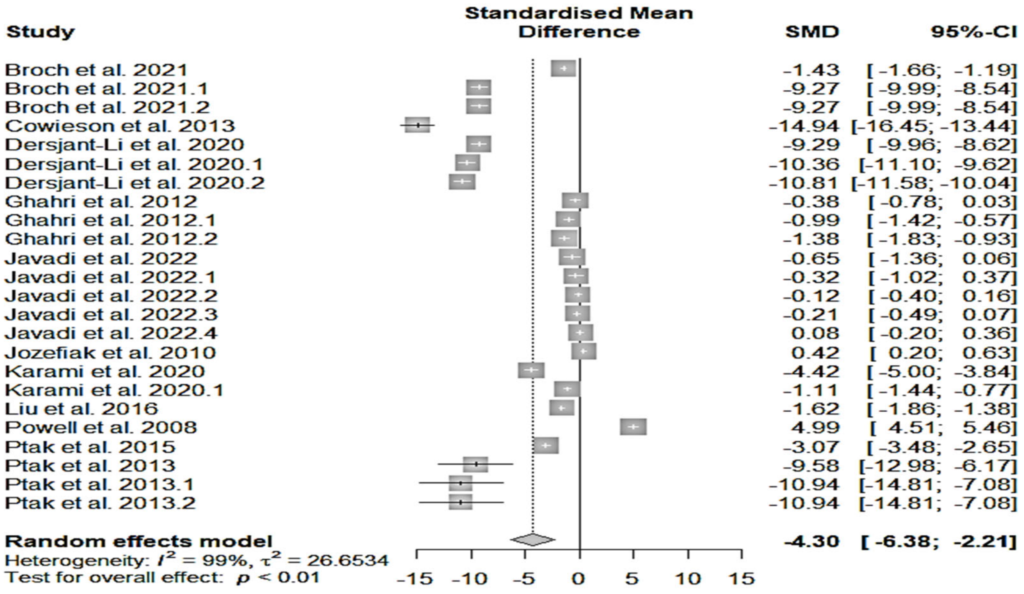


Figure S8. Forest plot of the effect size or standardized mean difference and 95% confidence interval of phytase on grower-finisher broilers' feed conversion ratio (FCR). The solid vertical black line represents the mean difference between zero and no effect. Points to the left of the solid vertical black line represent a reduction in FCR, while points to the right indicate an increase in FCR.

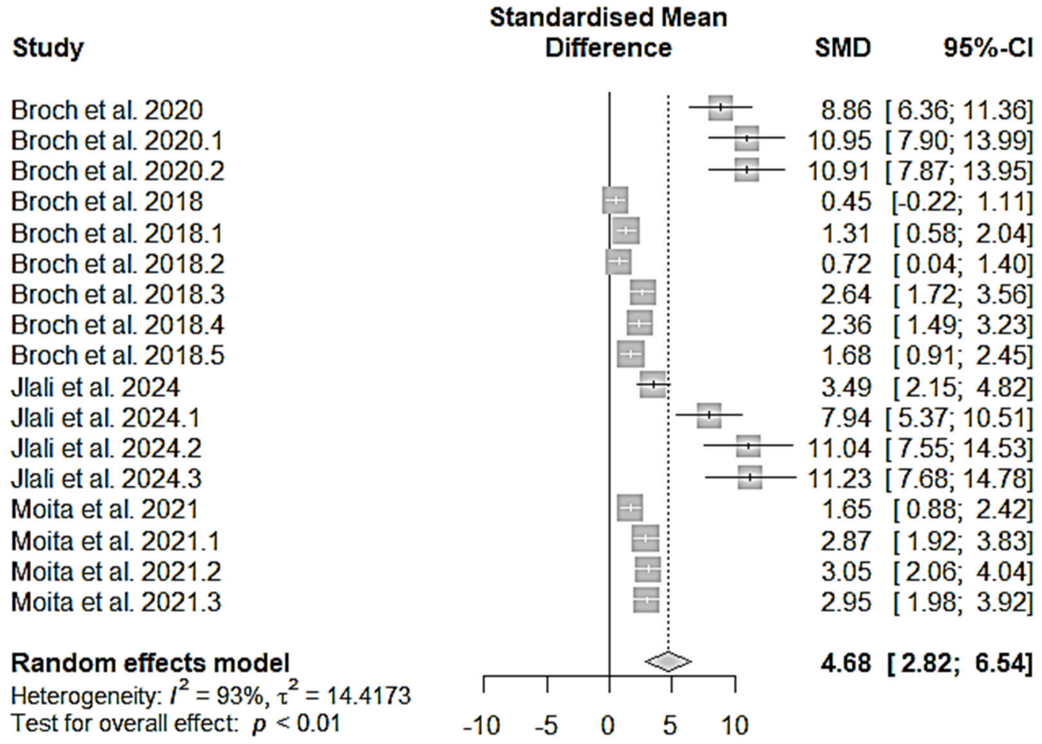


Figure S9. Forest plot of the effect size or standardized mean difference and 95% confidence interval of phytase on starter broilers' bone breaking strength (BBS). The solid vertical black line represents the mean difference between zero and no effect. Points to the left of the solid vertical black line represent a reduction in BBS, while points to the right indicate an increase in BBS.

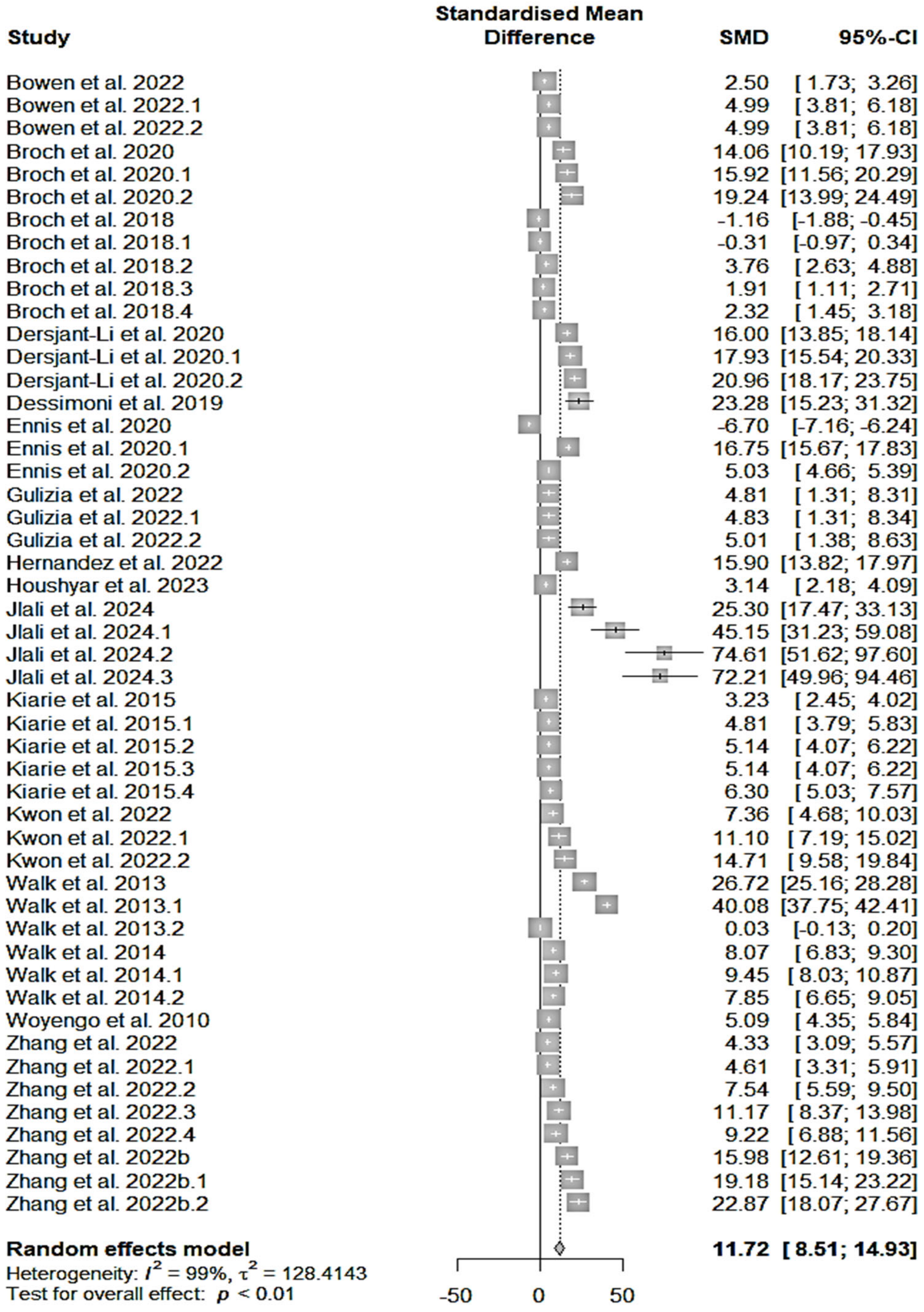


Figure S10. Forest plot of the effect size or standardized mean difference and 95% confidence interval of phytase on starter broilers' tibia ash (TA). The solid vertical black line represents the mean difference between zero and no effect. Points to the left of the solid vertical black line represent a reduction in TA, while points to the right indicate an increase in TA.

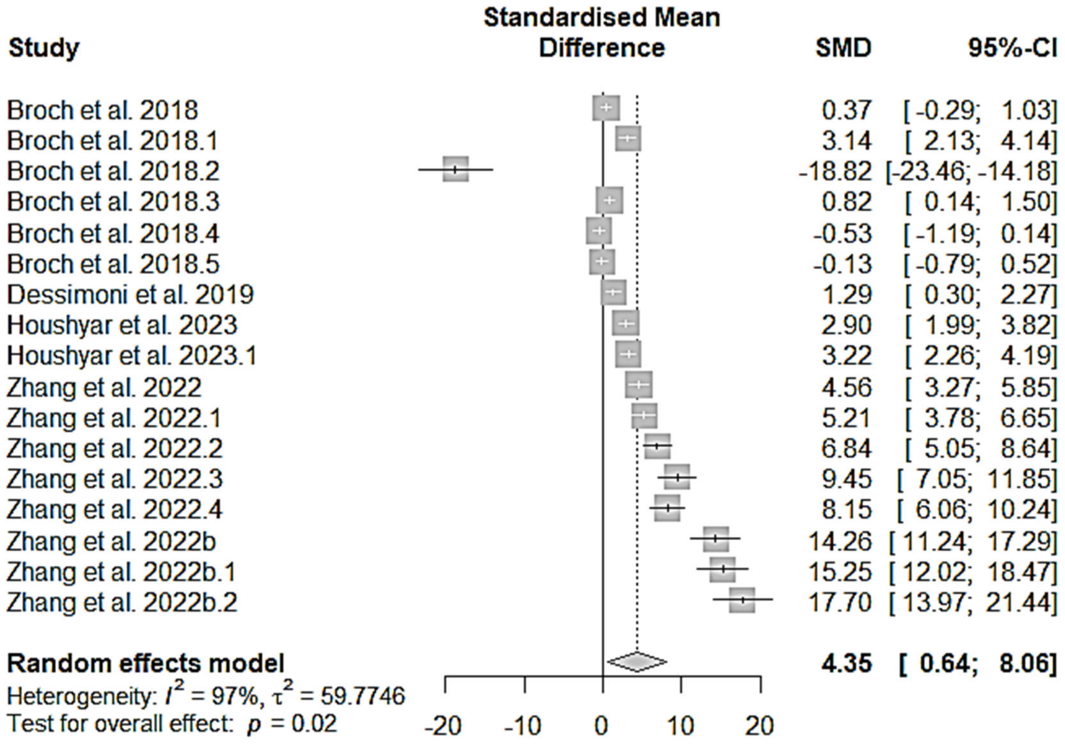


Figure S11. Forest plot of the effect size or standardized mean difference and 95% confidence interval of phytase on starter broilers' tibia calcium (TCa). The solid vertical black line represents the mean difference between zero and no effect. Points to the left of the solid vertical black line represent a reduction in TCa, while points to the right indicate an increase in TCa.

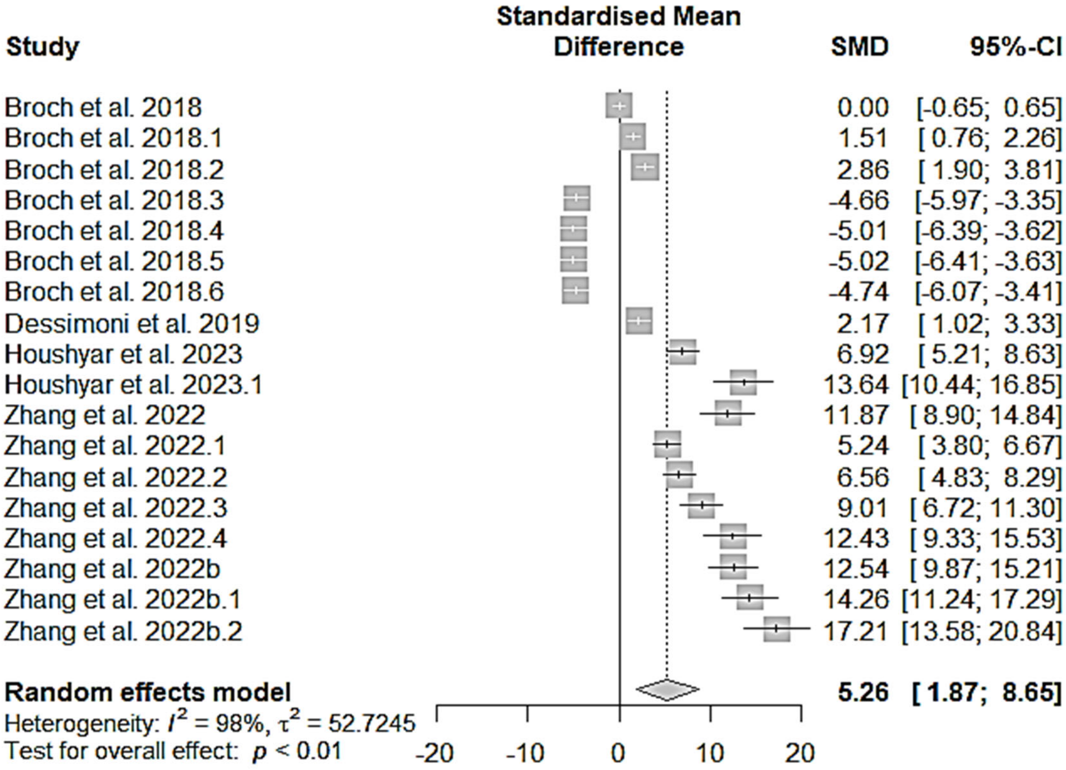


Figure S12. Forest plot of the effect size or standardized mean difference and 95% confidence interval of phytase on starter broilers' tibia phosphorus (TP). The solid vertical black line represents the mean difference between zero and no effect. Points to the left of the solid vertical black line represent a reduction in TP, while points to the right indicate an increase in TP.

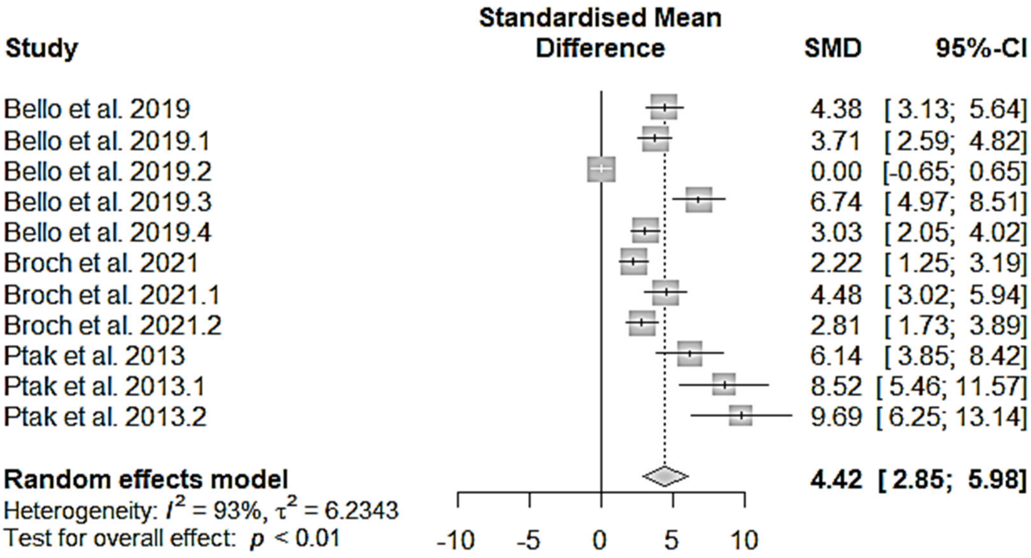


Figure S13. Forest plot of the effect size or standardized mean difference and 95% confidence interval of phytase on grower-finisher broilers' bone breaking strength (BBS). The solid vertical black line represents the mean difference between zero and no effect. Points to the left of the solid vertical black line represent a reduction in BBS, while points to the right indicate an increase in BBS.

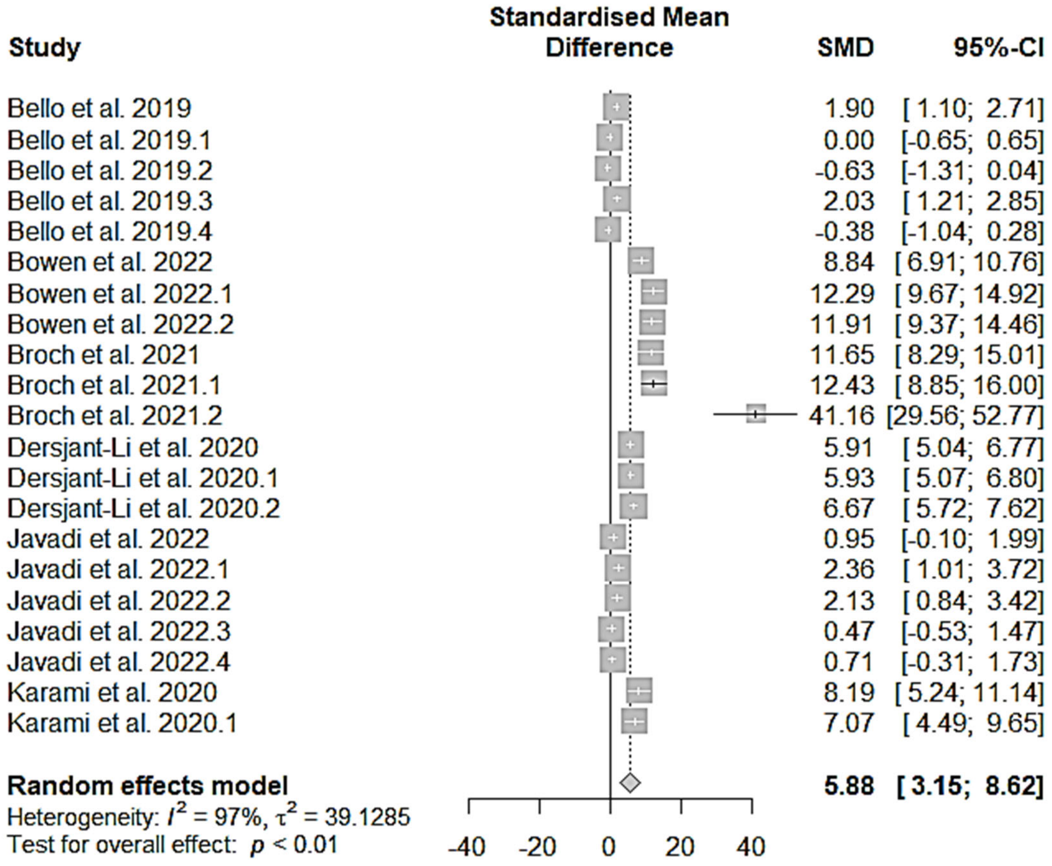


Figure S14. Forest plot of the effect size or standardized mean difference and 95% confidence interval of phytase on grower-finisher broilers' tibia ash (TA). The solid vertical black line represents the mean difference between zero and no effect. Points to the left of the solid vertical black line represent a reduction in TA, while points to the right indicate an increase in TA.

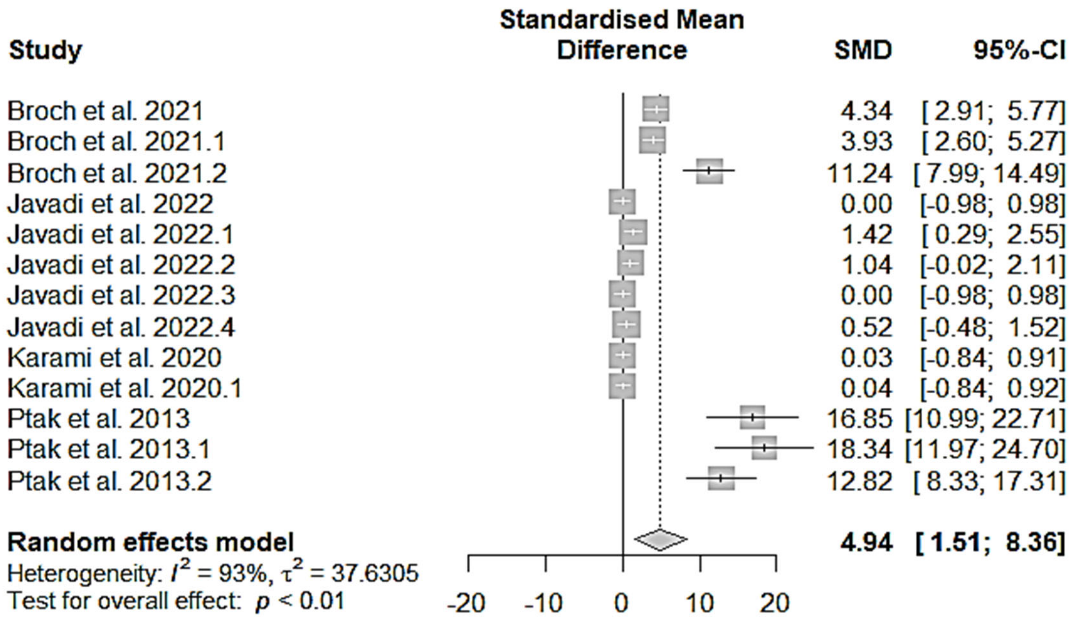


Figure S15. Forest plot of the effect size or standardized mean difference and 95% confidence interval of phytase on grower-finisher broilers' tibia calcium (TCa). The solid vertical black line represents the mean difference between zero and no effect. Points to the left of the solid vertical black line represent a reduction in TCa, while points to the right indicate an increase in TCa.

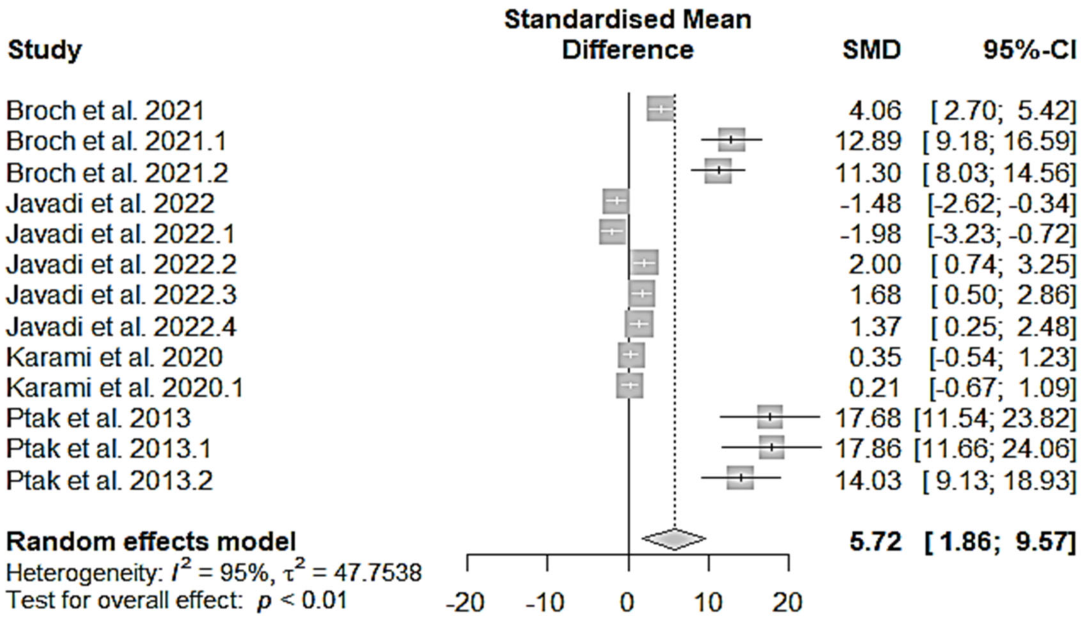


Figure S16. Forest plot of the effect size or standardized mean difference and 95% confidence interval of phytase on grower-finisher broilers' tibia phosphorus (TP). The solid vertical black line represents the mean difference between zero and no effect. Points to the left of the solid vertical black line represent a reduction in TP, while points to the right indicate an increase in TP.

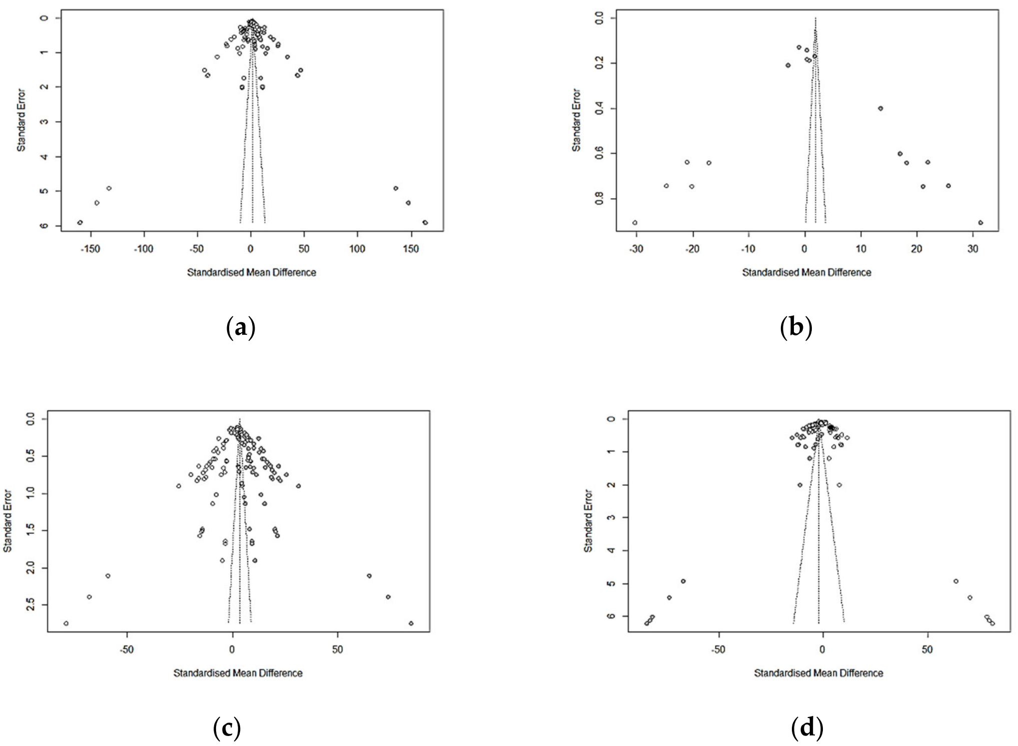


Figure S17. Trim and Fill funnel plot assessment for testing and adjusting publication bias on starter broilers' growth outcomes: (a) ADFI; (b) ADG; (c) BWG; (d) FCR.

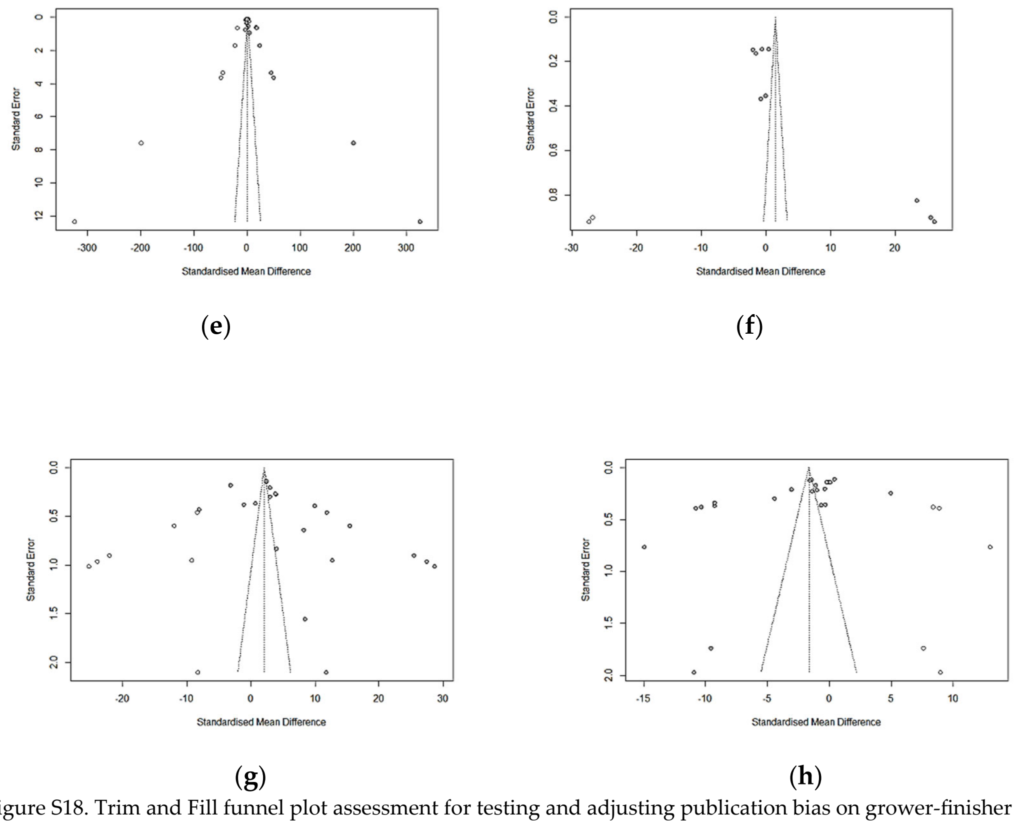


Figure S18. Trim and Fill funnel plot assessment for testing and adjusting publication bias on grower-finisher broilers' growth outcomes: (e) ADFI; (f) ADG; (g) BWG; (h) FCR.

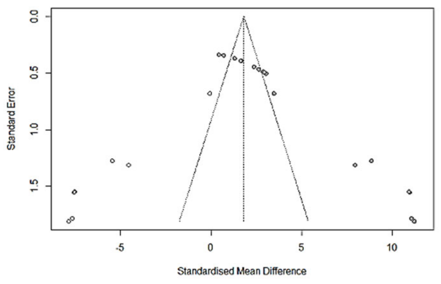


(**i**)

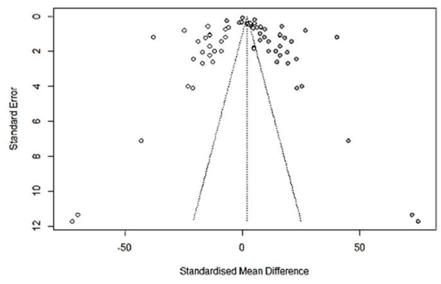


(**j**)

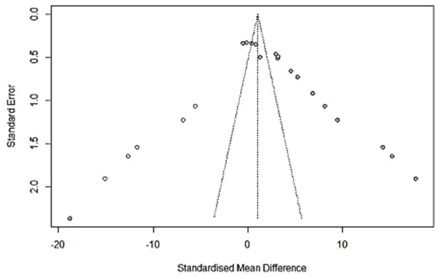


(**k**)

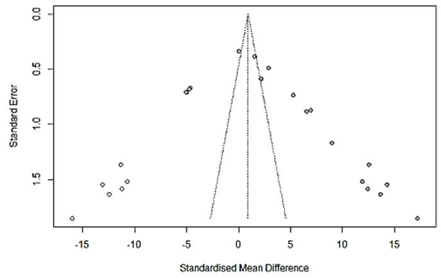


(**l**)

Figure S19. Trim and Fill funnel plot assessment for testing and adjusting publication bias on starter broilers' bone strength and mineralization outcomes: (i) BBS; (j) TA; (k) TCa; (l) TP.

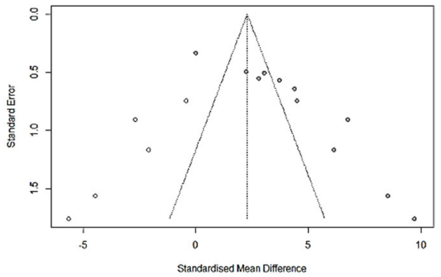


(**m**)

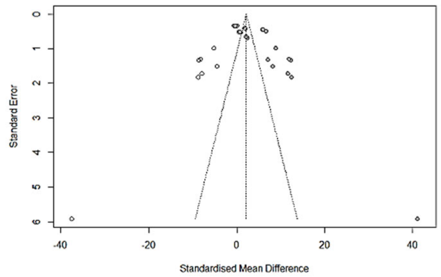


(**n**)

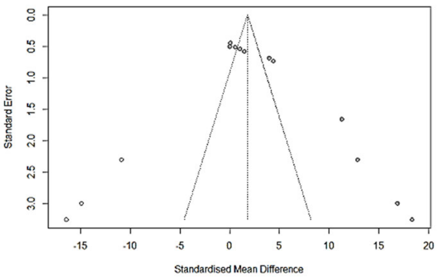


(**o**)

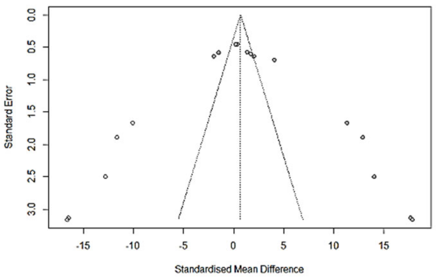


(**p**)

Figure S20. Trim and Fill funnel plot assessment for testing and adjusting publication bias on grower-finisher broilers' bone strength and mineralization outcomes: (m) BBS; (n) TA; (o) TCa; (p) TP.
